# Oral Phenotype of Singleton–Merten Syndrome: A Systematic Review Illustrated With a Case Report

**DOI:** 10.3389/fgene.2022.875490

**Published:** 2022-06-09

**Authors:** Margot Charlotte Riou, Muriel de La Dure-Molla, Stéphane Kerner, Sophie Rondeau, Adrien Legendre, Valerie Cormier-Daire, Benjamin P. J. Fournier

**Affiliations:** ^1^ Centre de Recherche des Cordeliers, UMRS 1138, Molecular Oral Pathophysiology, Université de Paris, INSERM, Sorbonne Université, Paris, France; ^2^ Dental Department, Reference Center for Oral and Dental Rare Diseases, AP-HP, Rothschild Hospital (ORARES), Paris, France; ^3^ Dental Faculty, Université de Paris, Paris, France; ^4^ INSERM U1163 Institut Imagine, Paris, France; ^5^ Department of Genetics, Necker Enfants Malades Hospital, Paris Descartes-Sorbonne Paris Cité University, Paris, France; ^6^ Laboratoire de Biologie Médicale Multisites Seqoia—FMG2025, Paris, France

**Keywords:** Singleton–Merten syndrome, rare diseases, oral physiopathology, genetics, type 1 interferonopathy

## Abstract

**Background:** Singleton–Merten syndrome type 1 (SGMRT1) is a rare autosomal dominant disorder caused by *IFIH1* variations with blood vessel calcifications, teeth anomalies, and bone defects.

**Aim:** We aimed to summarize the oral findings in SGMRT1 through a systematic review of the literature and to describe the phenotype of a 10-year-old patient with SGMRT1 diagnosis.

**Results:** A total of 20 patients were described in the literature, in nine articles. Eight *IFIH1* mutations were described in 11 families. Delayed eruption, short roots, and premature loss of permanent teeth were the most described features (100%). Impacted teeth (89%) and carious lesions (67%) were also described. Our patient, a 10-year-old male with Singleton–Merten syndrome, presented numerous carious lesions, severe teeth malposition, especially in the anterior arch, and an oral hygiene deficiency with a 100% plaque index. The panoramic X-ray did not show any dental agenesis but revealed very short roots and a decrease in the jaw alveolar bone height. The whole-genome sequencing analysis revealed a heterozygous *de novo* variant in *IFIH1* (NM_022168.4) c.2465G > A (p.Arg822Gln).

**Conclusion:** Confused descriptions of oral features occurred in the literature between congenital findings and “acquired” pathology, especially carious lesions. The dental phenotype of these patients encompasses eruption anomalies (delayed eruption and impacted teeth) and lack of root edification, leading to premature loss of permanent teeth, and it may contribute to the diagnosis. An early diagnosis is essential to prevent teeth loss and to improve the quality of life of these patients.

**Systematic Review Registration**: [https://www.crd.york.ac.uk/prospero/], identifier [CRD42022300025].

## Introduction

Singleton–Merten syndrome type 1 (SGMRT1, OMIM: 182250) is a rare autosomal dominant disorder associated with severe calcification of the ascending aorta and valves; acro-osteolysis widened medullary cavities of the distal limbs, scoliosis, and tooth anomalies (Singleton and Merten, 1973). The clinical characteristics of SMS showed a large variability of expressions. Psoriasis, muscular weakness, and glaucoma represent less frequently observed symptoms (Feigenbaum et al., 2013). Since its first description in 1973, few cases have been reported because of its low prevalence (1 < 1,000,000). A first missense heterozygous variant in the interferon-induced helicase C domain-containing protein 1 (*IFIH1*) gene was identified in three families (Rutsch et al., 2015). Since then, seven other pathogenic variants have been identified in patients with SGMRT1 (Bursztejn et al., 2015; de Carvalho et al., 2017; Takeichi et al., 2018; Vengoechea and DiMonda, 2020; Xiao et al., 2021; Hasegawa et al., 2022).

*IFIH1* encodes MDA5 protein, a member of the RIG-1-like receptor (RLR) family, which functions as a cytoplasmic pattern-recognition receptor recognizing viral double-stranded RNA (dsRNA) and secreted bacterial nucleic acids. Moreover, variants in the *DDX58* gene that encodes an RNA helicase were identified in individuals with similar phenotypes without dental anomalies (Jang et al., 2015). On the other hand, variants in the *IFIH1* gene were also causative of the Aicardi-Goutieres syndrome (AGS-7; OMIM 615846), an autosomal dominant inflammatory disorder characterized by severe neurologic impairment such as progressive encephalopathy, spastic paraplegia, and calcification of basal ganglia (Crow et al., 2015). The recent studies have also reported overlapping of the clinical findings of both syndromes (Bursztejn et al., 2015; Xiao et al., 2021; Hasegawa et al., 2022). Consequently, clinical diagnosis may be challenging.

Dental findings in SGMRT1 are described by OMIM as “delayed primary tooth exfoliation and permanent tooth eruption, truncated tooth root formation, early-onset periodontal disease, and severe root and alveolar bone resorption associated with dysregulated mineralization, leading to tooth loss” (SGMRT1, OMIM: 182250). Other authors describe “root dysplasia” (Takeichi et al., 2018), “primary dentition as hollow shells” (Vengoechea and DiMonda, 2020) or “severe dysplasia of root cementum and dentin” (Pettersson et al., 2017). Other features such as root defects seem unclear, and the frequency of their occurrence is not known. Moreover, craniofacial defects are reported but without precise description or prevalence.

We examined a patient with SGMRT1 and observed oral and craniofacial features. We, therefore, wondered whether the observed clinical manifestations were constant in previously reported cases. The purpose of this work was to summarize the oral signs associated with the SGMRT1 through a systematic review of the literature. We illustrated and compared it with a description of a clinical case. A more precise description of the clinical manifestations may allow an easier clinical diagnosis.

## Review of the Literature

### Methods

We conducted a systematic review of the literature using the PubMed database up until September 2021. To ensure its reproducibility, PRISMA guidelines were followed (Page et al., 2021), and the PRISMA flowchart was filled. The search term was “Singleton–Merten”. We aimed to precisely determine the oral clinical features of SGMRT1 patients with reported *IFIH1* variants. This review was registered with n°CRD42022300025.

### Inclusion and Exclusion Criteria

The inclusion criteria were as follows: articles in English or French and the phenotype in a human patient with an *IFIH1* mutation. The exclusion criteria were as follows: another language than English or French, animals or *in vitro* studies, narrative reviews, and lack of patient’s phenotype description, and Singleton–Merten patients with a variant in *DDX58*, or for whom the genetic cause has not been defined.

### Article Selection

The articles were evaluated for eligibility by title/abstract and then full-text screening using the Rayyan website (Ouzzani et al., 2016). Two reviewers assessed the articles separately. The recorded data were as follows: title/journal/date of publication of the article; authors; the number of patients, and their age/gender; mutation description and; description of dental phenotype with delayed eruption/carious lesions/short roots/premature loss of teeth/dental agenesis/low height of alveolar bone. We had chosen to group the different root manifestations/pathology/anomaly (resorption and lack of edification) under the term “short roots”.

### Visualization of Mutations

Using the Reference sequence of the *IFIH1* gene (NM_022168.4) and the associated protein sequence of melanoma differentiation-associated protein 5 (MDA5) (NP_071451.2), the domains in which the various mutations were located were determined using the Plot Protein website (Turner, 2013). For the conservation analysis, a multiple sequence alignment was generated using the following orthologs of human IFIH1: house mouse (NP_082111.2), zebrafish (NP_001295492.1), Norway rat (NP_001102669.1), pig (NP_001093664.1), tropical clawed frog (NP_031749133.1), chimpanzee (NP_°16805442.2), and coelacanth (NP_014348983.1).

### SEM Observation

A first permanent maxillary molar and a second primary mandibular molar were observed using SEM. The teeth were collected following the relevant guidelines related to research involving the patients’ samples in France (ethical approval n°19.11.04.64248, ORCELL). The samples were dehydrated using an ethanol gradient, before being thinly coated with gold using a Q15OR ES system (Quorum Technologies Ltd., East Sussex, UK). Then, it was observed using a SEM (TM3030 Tabletop Microscope, Hitachi) under few magnifications (from x1,5 k to x3,0 k) with a composite view.

## Results

### Article Selection

**FIGURE 1:**
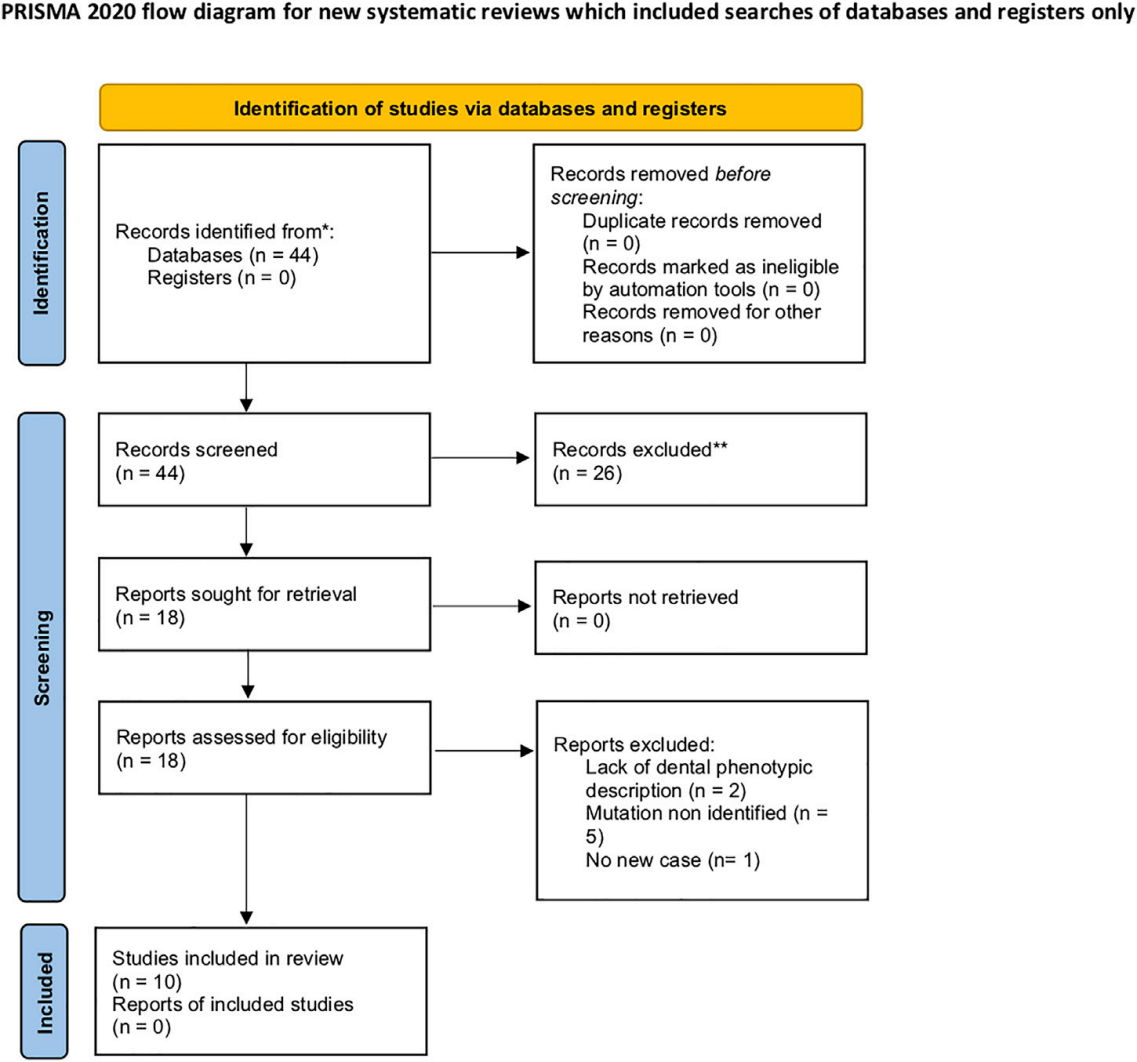
Flow chart of PRISMA.

A total of 44 articles were retrieved from the PubMed database. After full-text screening, 11 articles were included and analyzed (Figure 1), of which six were case reports and five were case series; two articles described the same patients: clinical description for the first one and mutation description for the second one. A total of 22 patients were described, 11 girls and 11 boys.

### Mutation Description

**FIGURE 2:**
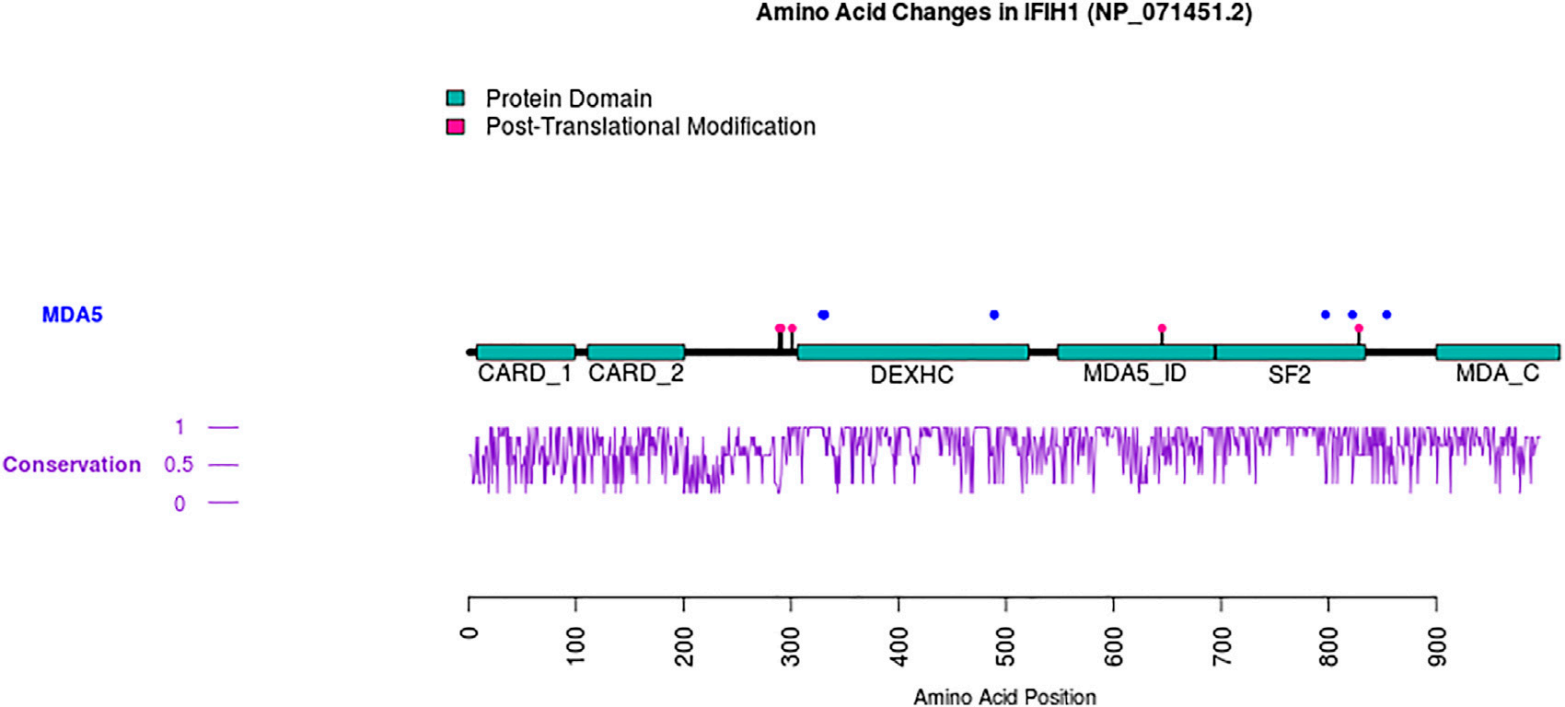
Visualization of the SMGRT1 mutations in MDA5. Plots of all disease-causing mutations in MDA5 associated to SGMRT1. The conservation score is between 0 and 1, with 0 indicating no other sequences matching the reference (*Homo sapiens* NP_071451.2) at the position, and 1 indicating all sequences matching the reference at that position. CARD1: caspase activation and recruitment domain found in MDA5, first repeat; CARD2: caspase activation and recruitment domain found in MDA5, second repeat; DEXHC: DEXH-box helicase domain of RLR-2; MDA5_ID: insert domain of MDA5; SF2: C-terminal helicase domain of the endoribonuclease dicer; MDA_C: C-terminal domain of melanoma differentiation-associated protein 5. The hotspot is represented in orange.

Eight *IFIH1* mutations were described (Table 1) in 11 families. One hotspot mutation seems to be evident (c.2465G > A) with nine patients through four families. To visualize the positions of protein domains and their amino acid boundaries’ positions, we used the RefSeq IFIH1, found on NCBI protein, NP_071451.2, containing 1025 amino acid residues (Figure 2). Five mutations were in helicase domain 1 (Hel1), two in helicase domain 2 (Hel2), and the last one in the pincer domain, which connects Hel2 and the C-terminal domain (CTD). In one article (Ghadiam and Mungee, 2017), an *IFIH1* mutation was reported but was neither described nor detailed.

**TABLE 1:** Mutation description.

Gene	Gene	Protein	Domain	Patient number (N; %)	Family number (N; %)	Article
IFIH1	c.986T > C	p.Leu329Pro	Hel1 domain*	1 (5%)	1 (8%)	(Vengoechea and DiMonda, 2020)
IFIH1	c.992C > G	p.Thr331Arg	Hel1 domain	2 (10%)	1 (8%)	(de Carvalho et al., 2017)
IFIH1	c.992C > T	p.Thr331Ile	Hel1 domain	3 (15%)	1 (8%)	(de Carvalho et al., 2017)
IFIH1	c.1465G > A	p.Ala489Thr	Hel1 domain	1 (5%)	1 (8%)	(Bursztejn et al., 2015)
IFIH1	c.1465G > T	p.Ala489Ser	Hel1 domain	1 (5%)	1 (8%)	(Xiao et al., 2021)
IFIH1	c.2390A > T	p.Asp797Val	Hel2 domain	1 (5%)	1 (8%)	(Hasegawa et al., 2022)
IFIH1	c.2465G > A	p.Arg822Gln	Hel2 domain*	9 (45%)	4 (33%)	(Feigenbaum et al., 2013; Rutsch et al., 2015; Pettersson et al., 2017)
IFIH1	c.2561T > A	p.Met854Lys	Hel2-CTD connection	1 (5%)	1 (8%)	(Takeichi et al., 2018)
IFIH1	NR	NR	NR	1 (5%)	1 (8%)	(Ghadiam and Mungee, 2017)
Total				20 (100%)	12 (100%)	

*The mutation associated domain was not notified in the article—NR: non-reported—all percentages have been rounded to the closet unit.

### Phenotype Description

The dental findings descriptions are summarized in Table 2. When signs were not reported, we specified (“not reported”).

**TABLE 2:** Patients’ dental descriptions.

Patient		Delayed eruption	Carious lesions	Short roots	Premature loss of teeth	Impacted tooth	Dental agenesis	Low height of alveolar bone	Mutation	Article	Evidence grade
Age	Gender										
9	M	Yes	No	Yes	Yes	-	Yes	Yes	c. 2390A > T	(Hasegawa et al., 2022)	4
41	M	-	-	Yes	Yes	No	-	No	c.1465G > A	(Bursztejn et al., 2015)	4
30	M	-	-	-	Yes	-	-	Yes	c.1465G > T	(Xiao et al., 2021)	4
28	F	Yes	No	Yes	Yes	Yes	No	Yes	c.2465G > A	(Pettersson et al., 2017)	4
-	F	Yes	-	Yes	Yes	Yes	-	-			
5	F	-	-	Yes	Yes	Yes	-	-		(Feigenbaum et al., 2013; Rutsch et al., 2015)	4
25	M	-	-	-	Yes	Yes	-	-			
4	M	Yes	Yes	Yes	Yes	-	No	Yes			
3	F	Yes	Yes	Yes	Yes	Yes	No	Yes			
3	M	Yes	-	Yes	Yes	Yes	No	-			
Child	M	-	-	-	Yes	-	-	-			
3	F	Yes	Yes	Yes	Yes	-	Yes	-			
7	F	Yes	-	Yes	-	Yes	-	Yes	c.2561T > A	(Takeichi et al., 2018)	4
30	F	-	-	-	-	Yes	-	-	c.986T > C	(Vengoechea and DiMonda, 2020)	4
9	F	Yes	-	Yes	Yes	-	-	-	c.992C > G	(de Carvalho et al., 2017)	4
47	M	Yes	-	Yes	Yes	-	-	-			
18	F	Yes	-	-	-	-	-	-	c.992C > T		
45	F	-	-	-	Yes	-	-	-			
27	F	-	-	-	Yes	-	-	-			
30	M	Yes	-	-	-	-	-	-	-	(Ghadiam and Mungee, 2017)	4

The most frequent dental findings were as follows: short roots, delayed eruption, and premature loss of permanent teeth (present in 100% of screened patients). The patients showed in addition impacted permanent teeth (89%), a decreased height of alveolar bone (86%), and carious lesions (67%). Two patients were described with dental agenesis (Table 3). However, oral data were absent in almost 50% of patients, and the most constant sign examined or reported was “premature loss of permanent teeth”.

**TABLE 3:** Oral and dental phenotypes of Singleton–Merten patients.

	Yes (%)	No (%)	NR (%)	% Among patients with oral examination (% yes)
Delayed eruption	12 (60%)	0 (0%)	8 (40%)	100
Carious lesions	3 (15%)	2 (10%)	15 (75%)	60
Short roots	11 (55%)	0 (0%)	9 (45%)	100
Premature loss of permanent teeth	16 (80%)	0 (0%)	4 (20%)	100
Impacted teeth	8 (40%)	1 (5%)	11 (55%)	89
Dental agenesis	2 (10%)	4 (20%)	14 (70%)	33
Low height of alveolar bone	6 (30%)	1 (5%)	13 (65%)	86

N: number of concerned patients; NR: non-reported.

The patient described by Takeichi et al. (2018) showed a different oral phenotype/manifestation. On the X-rays, we observed that none of the primary and permanent teeth were erupted, while all the dental germs were visible in the jawbones.

## Case-Report

**FIGURE 3:**
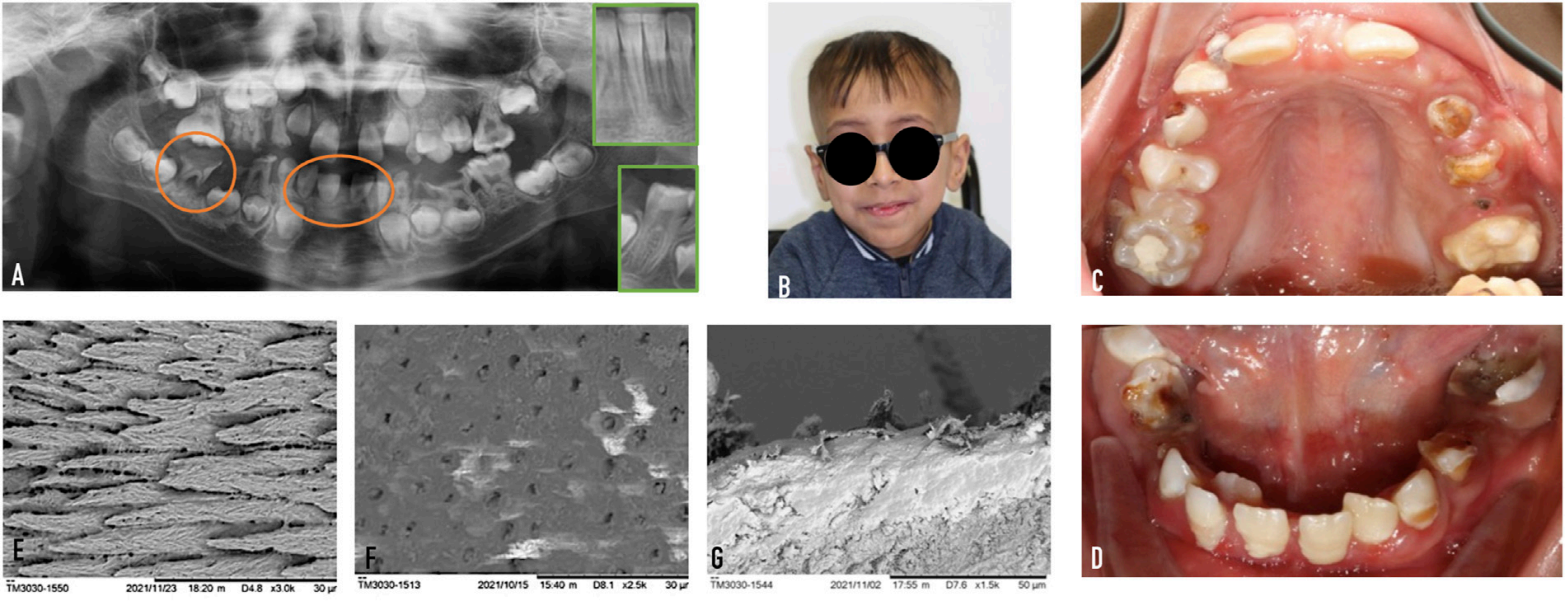
Case report. **(A)** Orthopantomogram X-ray of our 10-year-old Singleton-Merten patient. In orange, a first permanent molar (tooth n°46) and central permanent mandibular incisors (teeth n° 31–41) with short roots were highlighted. To compare, a healthy patient’s teeth are shown in a green insert. **(B)** Photography of the patient. **(C,D)** Intra-oral photographies of the maxillary and the mandibular arch. **(E)** First molar enamel. **(F)** Second temporary molar dentin. **(G)** First molar cement.

A 10-year-old child was referred to the Reference Centre of Oral and Dental Rare Diseases at Rothschild Hospital (AP-HP). Written informed consent was obtained from the patient and his legal guardian mother for the publication of any potentially identifiable images or data included in this article. The patient experienced pain due to numerous carious lesions, associated with dental and jawbone anomalies visible on the panoramic radiograph (Figure 3). He was the third child of a sibship of four healthy children from a non-consanguineous union. He had recently arrived in France, for medical reasons. According to his mother, he walked until he was 1 year old and then progressively developed walking difficulties and muscle weakness requiring a wheelchair at 10 years of age. He presented cutaneous xerosis and ophthalmologic glaucoma. No intellectual disability was noticed.

We observed dysmorphic facial features: fine and space hair, cranial malformation as trigonocephaly with a triangular face, discrete hypertelorism, long arched eyebrow, and low set-ears. He had clubfeet, joint retractions, and scoliosis. The weight and height were below—2SD. Intra-oral examination revealed multiple caries, severe teeth malposition, especially in the anterior arch, and oral hygiene deficiency with a 100% plaque index (Figure 3). On X-ray examination, we did not find any dental agenesis. All the germs of the permanent teeth were visible, including the third permanent molars. The examination revealed the presence of thin roots in primary teeth and undeveloped roots in permanent teeth. The roots were shortened beyond the first root third despite the closure of the dental apices. Almost all primary teeth and permanent molars presented extensive-stage caries with abscesses (ICDAS codes 5 and 6, RC 6). The teeth morphology showed a bulbous-shaped crown, with normal pulp chamber volume. Teeth were mobile (mobility II-III). We observed a moderate to severe gingival inflammation: bright surface inflammation, erythema, edema and/or hypertrophy of gingiva, and some spontaneous bleedings. We did not observe deep pockets or recessions. The panoramic X-ray revealed a reduction in the alveolar bone height. The whole-genome sequencing analysis revealed a heterozygous *de novo* variant in the *IFIH1 gene* (NM_022168.4) c.2465G > A (p.Arg822Gln).

SEM analysis showed that neither enamel nor dentin defects were associated with SGMRT1, and normal cementum was present. Indeed, we observed normal enamel prisms, dentin tubules, and a visible cementum layer.

## Discussion

The oral phenotype of Singleton–Merten syndrome was confusing in the literature. The most frequent anomaly concerns root, dental eruption, and premature tooth loss. In this systematic review, 100% of the case reports described “short roots” and “premature loss of permanent teeth.” The short root is a quantitative tooth anomaly easily recognizable on X-rays. Regarding the X-rays available within articles, the short roots were mostly concerned with permanent dentition (primary teeth show long and fine roots). The shortness of the roots may result from congenital root deficiency during root formation or in the radicular resorption process. Root resorption is defined as a progressive loss of dentin and cementum through the continued action of osteoclastic cells (Fuss et al., 2003). In this literature review, the authors described indifferently “short roots” (Pettersson et al., 2017), “loss of root tooth structure,” and “aggressive resorptive process” (Feigenbaum et al., 2013). We analyzed the available X-rays to clarify these findings. We did not find any radiographic signs of resorption, such as an enlargement of the root canal, an asymmetric bowl-shaped radiolucency, or an asymmetric loss of root, as described in classical root resorption (Patel and Saberi, 2018). Conversely, we observed closed root apices. We suggested that the root defects observed in SGMRT1 patients are an impairment in root elongation more than in a resorption phenomenon. This lack of root development seems to be the cause of the premature loss of permanent teeth, as described by the majority of the authors (Feigenbaum et al., 2013; Bursztejn et al., 2015; Rutsch et al., 2015; de Carvalho et al., 2017; Pettersson et al., 2017; Xiao et al., 2021; Hasegawa et al., 2022) and as observed in the patient. Teeth root anomalies are also observed in radicular dentin dysplasia and Fraser syndrome. We can discriminate the SGMRT1 patients from radicular dentin dysplasia because of the lack of pulp obliteration and from Fraser syndrome because of the lack of short roots in primary teeth (de La Dure-Molla et al., 2015; Luder, 2015).

When reported, “delayed eruption” was observed in 100% of the patients, and “impacted teeth,” in 89%. Delayed eruption and impacted teeth can be difficult to discriminate. A normal eruption occurred over a period of 2 years, and a delayed eruption is defined by a tooth eruption more than 2 SD beyond the mean eruption age (de La Dure-Molla et al., 2019). The eruption must be tracked over time to determine if teeth are impacted or had just a delayed eruption. In this review, the patients were often too young, and this finding must be reevaluated in adults. So we cannot conclude if the tooth eruption has been delayed or failed. In our patient, no impacted tooth was noticed. However, three patients had no tooth eruption (Singleton and Merten, 1973; Takeichi et al., 2018). For the patients described by Singleton and Merten, no genetic analysis was performed; for the second report, the patient was diagnosed with SMS and AGS-7. We concluded that the pathology of an eruption occurring in SMS must be confirmed by a refined analysis comparing the dental age and civil age.

Furthermore, a great diversity of features appeared in the various case reports, such as deficiency of alveolar bone and carious lesions. Several SGMRT1 patients presented a deficiency of alveolar bone growth. Osteopenia is often reported in SGMRT1 patients’ limbs, which might be also found in jawbones. The alveolar bone growth is directly linked to root development and teeth eruption. The absence of root elongation and the premature loss of the teeth may therefore lead to this defective bone.

Our patient was in mixed dentition. The remaining primary teeth had thin roots with normal length, and all erupted permanent teeth had short roots and mobility. Clinical examination and SEM observation did not reveal any dental tissue (enamel, dentin, and cementum) anomalies. A radiological exam was necessary to identify the root anomalies. Here, we reported a heterozygous *de novo* variant in *IFIH1* c.2465G > A (p.Arg822Gln). This variant has been previously described in Singleton–Merten syndrome in nine patients through four families (Feigenbaum et al., 2013; Rutsch et al., 2015; Pettersson et al., 2017). It is the most prevalent reported hotspot. Until now, all reported variants are missense with a gain-of-function effect and an enhanced expression of type I interferon-stimulated genes (Rice et al., 2020).

The role of *IFIH1* is still poorly understood, and a systematic description of dental signs in patients with an *IFIH1* mutation should help improve the understanding of its function in odontogenesis. IFIH1 gain-of-function is associated with dysregulation of mineralization genes in pulp cells (Lu et al., 2014). However, its role in odontogenesis, root edification, periodontium development, and homeostasis is yet to be explored. *IFIH1* plays a role in response to viral infection and then participates in nuclear factor kappa-B (NFkB) and interferon regulatory factors (IRF) activation. Amazingly, the SGMRT1 patients do not present any reported higher risks of viral infections. The only infectious feature reported in SGMRT1 patients was dental caries. It is an infectious disease linked to bacteria (Chardin et al., 2006). Finally, confused descriptions on oral features occurred in the literature between congenital findings and “acquired” pathology in the SGMT1 patients. Indeed, caries can be explained by oral hygiene deficiency and painful or mobile teeth. It may also be attributed to the muscular weakness or glaucoma exhibited by some SGMRT1 patients.

## Conclusion

The dental anomalies observed in SGMRT1 seem to affect mainly permanent teeth with variable expressivity. Two main features appeared constant: tooth permanent short roots with closed apex inducing premature loss and tooth eruption defects (delayed or potentially impacted teeth). The pathological exfoliation of the permanent teeth could be considered a pathognomonic and could help in diagnosis. A more systematic description of the dental phenotype with well-defined diagnosis criteria is necessary to better understand the dental phenotype in these patients. Also, an oral evaluation and a follow-up by a dental surgeon are recommended. A fundamental research is needed to understand the dental root formation and tooth eruption and the *IFIH1* impact on these processes.

## Data Availability

The original contributions presented in the study are included in the article/supplementary material; further inquiries can be directed to the corresponding author.
